# Seasonal dynamics modifies fate of oxygen, nitrate, and organic micropollutants during bank filtration — temperature-dependent reactive transport modeling of field data

**DOI:** 10.1007/s11356-020-11002-9

**Published:** 2020-11-05

**Authors:** Isolde S. Barkow, Sascha E. Oswald, Hermann-Josef Lensing, Matthias Munz

**Affiliations:** 1 Institute of Environmental Science and Geography, University of Potsdam, Karl-Liebknecht-Str. 24-25, 14476 Potsdam, Germany; 2 Department of Geotechnical Engineering, Federal Waterways Engineering and Research Institute (BAW), Kussmaulstraße 17, 76187 Karlsruhe, Germany

**Keywords:** Bank filtration, Aerobic and anaerobic conditions, Pharmaceuticals and personal care products, Reactive transport modeling, Degradation

## Abstract

Bank filtration is considered to improve water quality through microbially mediated degradation of pollutants and is suitable for waterworks to increase their production. In particular, aquifer temperatures and oxygen supply have a great impact on many microbial processes. To investigate the temporal and spatial behavior of selected organic micropollutants during bank filtration in dependence of relevant biogeochemical conditions, we have set up a 2D reactive transport model using MODFLOW and PHT3D under the user interface ORTI3D. The considered 160-m-long transect ranges from the surface water to a groundwater extraction well of the adjacent waterworks. For this purpose, water levels, temperatures, and chemical parameters were regularly measured in the surface water and groundwater observation wells over one and a half years. To simulate the effect of seasonal temperature variations on microbial mediated degradation, we applied an empirical temperature factor, which yields a strong reduction of the degradation rate at groundwater temperatures below 11 °C. Except for acesulfame, the considered organic micropollutants are substantially degraded along their subsurface flow paths with maximum degradation rates in the range of 10^−6^ mol L^−1^ s^−1^. Preferential biodegradation of phenazone, diclofenac, and valsartan was found under oxic conditions, whereas carbamazepine and sulfamethoxazole were degraded under anoxic conditions. This study highlights the influence of seasonal variations in oxygen supply and temperature on the fate of organic micropollutants in surface water infiltrating into an aquifer.

## Introduction

Since the last years, the occurrence of organic micropollutants (OMPs) is increasing in rivers and groundwater. Personal care products (PPCP) like pharmaceuticals or food additives are widespread and detectable in water bodies (Karam and Nicell 1997; Loos et al. 2010; Sui et al. 2015). It was already found out by Heberer et al. (1998) that concentrations of pharmaceuticals occur in the microgram per liter range for surface waters in Berlin, Germany. Estrogens, analgesics, antibiotics, or antiepileptics are particularly common in bank filtrate and groundwater in nanograms per liter levels, and these drug concentrations also occur worldwide (Heberer et al. 2008; Loos et al. 2010; Zhang et al. 2016). Since the water consumption is continuously rising and water resources are affected by human activity, use, and contamination, techniques for a good water quality become more important (Hancock 2002; McEachran et al. 2016).

Bank filtration (BF) is a common technique to improve water quality (Huntscha et al. 2013; Hamann et al. 2016). During BF microorganisms, for example, fungi and bacteria can degrade OMPs, polymerize, or remove them from the water by accumulating them in the biomass. So, water from lakes and rivers can be purified through a bank filtration passage. In addition, high plume concentrations can be reduced and delayed by dispersion, dilution, and sorption to the solid phase. Retention and dilution have less influence than degradation on the purification of the infiltrated surface water (Huntscha et al. 2013).

The soil and solid phase contain a large number of microorganisms, whose growth conditions are decisive for the degradation rate of OMPs. The efficiency of microbial degradation is influenced by the residence time of the bank filtrate, redox conditions, substrate availability, and temperature. Microorganisms use organic carbon compounds dissolved in water or present as particulate substances in the substrate to obtain energy for cell metabolism to sequentially reduce (consume), e.g., O_2_, NO_3_^−^, Mn^4+^, Fe^3+^, and SO_4_^2−^ (Lensing et al. 1994; McMahon and Chapelle 2008). Organic matter is used as a primary substrate but microorganisms can also be specialized to other carbon sources like OMPs (Lingens et al. 1985; Arnosti et al. 1998).

Several studies observed an increase in microbial activity and thus in OMP degradation rates with increasing temperatures. Burke et al. (2018) investigated the degradation rate constant of OMPs, e.g., valsartan acid in a column study by averaged 21 °C. But if the temperature was specified at different levels, it was found that microbial activity and biodegradation increased with temperature (Burke et al. 2014; Burke et al. 2017). Munz et al. (2019) showed that in situ degradation rates of OMPs, e.g., diclofenac in BF system, substantially varied for temperature changes between 5 and 20 °C.

Differences in labor and in situ studies regarding approach conditions and determined parameters lead to less comparability. In addition, in most batch and column laboratory studies, temperature is not specified. Additionally, it was found that the majority of degradation rates for several compounds varied over more than three orders of magnitude in different studies (Greskowiak et al. 2017). Based on spatial and temporal variation of observed OMPs, in situ approaches were applied to determine averaged, first-order degradation rates for actual redox conditions and water temperatures (Huntscha et al. 2013; Henzler et al. 2014). Considering attenuation of OMPs, labor and field studies have differences in approach conditions, e.g., specific degradation conditions. As an example, for carbamazepine, possible degradation under oxic and anoxic conditions was described (Massmann et al. 2009; Burke et al. 2018; Munz et al. 2019). Additionally, only a few studies exist which determined the degradation behavior of valsartan. Munz et al. (2019) analytically determined an averaged first-order degradation rate for valsartan. For this reason, this study is also intended to provide further knowledge about OMP degradation at real sites, regarding the maximum rate constants for different OMPs.

In order to accurately determine the effect of maximum degradation rate constant, preferential aquifer conditions (oxic/anoxic) and temperature on the fate of OMP during bank filtration reactive transport models were set up and calibrated against measured OMP concentrations. Only few studies determine temperature-dependent in situ rates. Greskowiak et al. (2006) simulated phenazone degradation, inhibited by anoxic conditions, under consideration of varying water temperatures using an empirical temperature relationship, which is multiplied to the maximum consumption rate. Sharma et al. (2012) investigated the redox zonation under temperature influence for a BF system at the Rhine River. Changes in water temperature and residence times were determined as the main factors for changes in redox zonation. They adopted the implementation of temperature dependency from microorganisms’ catalyzed reactions from Greskowiak et al. (2006). Engelhardt et al. (2013b) quantified transport and degradation of iomeprol and transformation products under transient conditions at field scale. Henzler et al. (2016) investigated the seasonality of redox zonation for a managed aquifer recharge system at the Lake Tegel in Berlin and found that the observed concentrations of nitrate and oxygen could be reproduced when considering temperature dependence of the redox reaction kinetics. However, reactive transport simulations to investigate the fate and behavior for various OMPs, considering redox conditions as well as the temperature dependence of the reaction kinetics, for bank filtration systems are still rare.

The present study aims to simulate seasonal varying oxygen and nitrate consumption as the basis for a multispecies reactive transport of several OMPs under consideration of varying water temperatures. We create a 2D model of a cross section for a bank filtration site at a nearby waterworks of Potsdam, Germany. The model was calibrated against measurement data for a period of over one and a half years and thus provides filed scale, in situ, estimates of degradation rate constants, and their dependence on aquifer temperature. The investigated OMPs are food sweeteners (acesulfame) or are part of the personal and pharmaceutical care products group (phenazone, diclofenac, valsartan, sulfamethoxazole, and carbamazepine). These OMPs are known to occur in surface water and groundwater and to preferentially degrade under different redox conditions.

## Materials and methods

### Study site

**Fig. 1:**
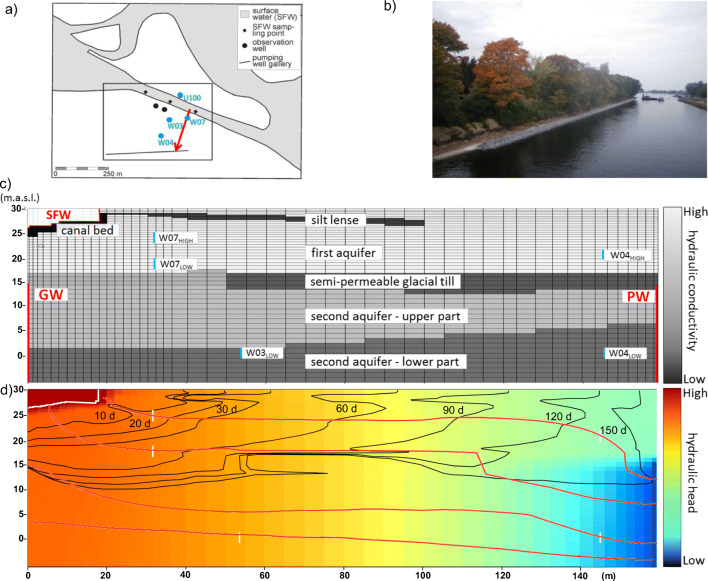
(a) Plan view of the study site with the vertical cross section (red line) and considered observation wells (blue dots). (b) Photograph of the Nedlitzer Durchstich towards lake Weisser See showing the southern canal banks. (c) Model domain, grid discretization, and approximate position of the observation wells (blue lines). The Dirichlet boundary conditions of the numerical model are indicated by red lines representing the hydraulic head in the canal (SFW), in the ambient groundwater (GW), and at the pumping well (PW). (d) Distribution of residence time isolines, selected flow lines, and hydraulic heads in August 2017

The study site is located in Potsdam, Germany, where the pumping activity of the communal waterworks induces bank filtration at the southern shore of the canal “Nedlitzer Durchstich,” which is a navigable shortcut between two lakes belonging to the Havel river system (Fig. 1 a and b) and the city of Berlin being located upstream. Continuous water extraction leads to yearly averaged dropdown in groundwater level from the surface water (29.4 m above sea level, standard deviation (std: 0.10 m)) to the well gallery (22.4 m.a.s.l., std: 0.07 m) of about 7 m. The inflow is affected by bank filtration from the canal and the hydraulic conditions in the deeper aquifer as a result of the hydraulic gradient in the direction of the pumping wells, with temporally varying groundwater levels. The canal water temperature ranged from 0.6 °C in January 2017 to 22.9 °C in August 2017. For observation points close to the shoreline, water temperature shows a stronger seasonal variation than in more distant wells. The average groundwater temperature was about 12.4 °C. Bank filtration occurs in the quaternary, sandy uppermost two aquifers which extend from approximately 36 m.a.s.l to 5 m below sea level and are divided by a semipermeable glacial till layer located between 15 m and 17 m a.s.l. (Fig. 1 c). A silt layer from the Weichsel cold period is fragmentary due to periglacial processes and erosion so that silts occur as lenses in the area and the local layer is implemented as a thin zone in the upper part of the first aquifer, according to Wang et al. (2020).

The spatial and temporal variations in groundwater flux, as well as temperature and partly redox conditions at this bank filtration site, were studied already by Wang et al. (2020) and Munz et al. (2019). According to Munz et al. (2019), the hydrological system is seen to be fairly stable in time with a high share of bank filtrate ranging between 61% (W04_HIGH_) and 100% (W07_HIGH_) and average subsurface residence times ranging between 18 d (W07_HIGH_) and 438 d (W04_LOW_).

This study focuses on a 2D vertical cross section, almost perpendicular to the canal and along a groundwater flow line towards a drinking water production well of the nearby waterworks. Five groundwater observation wells that are located along this transect were considered in the interpretation (W07_HIGH & LOW_, W03, W04_HIGH & LOW_) (Fig. 1 a). At W07 and W04, two observation wells were placed together with different filter screen elevations (Fig. 1 c). Hydraulic heads (electric contact gauge with an accuracy of ± 0.01 m), temperature (WTW-IDS with an accuracy of ± 2%), and dissolved oxygen (WTW-IDS ± 1.5%) were measured every 4 weeks from December 2015 until August 2017, during representative groundwater sampling campaigns. The collected water samples were analyzed for major ions (Dionex DX-120 & Thermo iCAP 6300 Duo) and selected organic micropollutants (HPLC-MS/MS). The limits of detection (LOD) were 6.25 × 10^−7^ mol L^−1^ for dissolved oxygen, and 1.21 × 10^−5^ mol L^−1^ for NO_3_^−^. Details of the water sampling and analysis can be found in Munz et al. (2019). In our study, acesulfame (LOD = 4.97 × 10^−11^ mol L^−1^), diclofenac (3.4 × 10^−11^ mol L^−1^), phenazone (5.31 × 10^−11^ mol L^−1^), sulfamethoxazole (4.0 × 10^−11^ mol L^−1^), carbamazepine (4.2 × 10^−11^ mol L^−1^), and valsartan (4.6 × 10^−11^ mol L^−1^) were considered, which strongly vary in their detected concentrations in the groundwater observation boreholes in space and time. Except for acesulfame, their degradation behavior was found to substantially depend on redox conditions and temperature (Engelhardt et al. 2013b; Munz et al. 2019).

### Model setup

#### Water flow and heat transport model

We applied a transient 2D water flow, heat, and reactive transport model to simulate the hydrochemical processes at the bank filtration site. The transect was extracted from a calibrated 3D flow and heat transport model of the study area (Wang et al. 2020) following a flow line from the canal towards a pumping well (Fig. 1 a). The geological structure of this 3D model was derived from about 145 borehole profiles whereby most of the drilling locations are placed between the canal bank and pumping wells. The uppermost two aquifers are separated by a fractured aquitard which was represented as two zones with slightly different hydraulic conductivities (Fig. 1 c, Table 2). The spatial dimensions of the selected model transect was 159.2 m in the horizontal and 30 m in the vertical direction. Initial horizontal hydraulic conductivities and anisotropy ratios between horizontal and vertical hydraulic conductivity were set according to the large scale, 3D flow, and heat transport modeling of Wang et al. (2020) but were subject to recalibration (cp. “Model platform and calibration procedure”). The specific yield and the effective porosity in the aquifers were both set to 0.23, but only to 0.06 in the remaining low permeable layers. The longitudinal dispersivity α_L_ was estimated to be about 1 m for the upper aquifer; otherwise, a value of 0.5 m has been established in the model and the transversal dispersivities are one-tenth of them, adapted from Wang et al. (2020) and Huang et al. (2003).

The horizontal cell width was set to two meters between the left-hand side of the transect and W07 and increases continuously up to 5 m towards W04 until it decreases again towards the right-hand side of the transect (Fig. 1 c). In the vertical direction, the cell length ranges from 0.3 m close to the canal to 1 m in the second aquifer. The chosen cell sizes provided an adequate spatial resolution to capture the naturally occurring gradients in the hydraulic head, temperature, and solute species concentrations.

The transient simulation was carried out from the 15 December 2015 to 1 August 2017. To determine realistic initial conditions, a spin-up period of about 1 year with the boundary conditions from January to December 2016 was added to the simulations. The boundary conditions of the transient simulation were updated about every month according to the sampling intervals (for details see Munz et al. 2019).

Measurements of the hydraulic heads and temperatures from surface water and the ambient groundwater were taken as Dirichlet boundary at the canal bottom (SFW in Fig. 1 c) and the left-hand side of the 2nd aquifer (GW in Fig. 1 c), respectively. To represent the ambient groundwater, measurements from the observation point U100 were taken (Fig. 1 a). The transient hydraulic heads for the outflow boundary condition at the pumping well (PW in Fig. 1 c) correspond to the simulated results from Wang et al. (2020). For temperatures and concentrations, default no-dispersive flux boundaries of PHT3D were used for this outflow boundary. The upper and lower boundaries of transect are implemented as no-flow boundaries.

Heat transport in the aquifer is governed by heat advection, heat conduction, dispersion through the fluid and aquifer sediment, and heat exchange between the aqueous phase and the aquifer sediment. The retardation (R) of the thermal front in comparison with a conservative tracer can be defined as (e.g., Zheng and Wang 1999):

1 $$ R=1+\frac{\rho_b}{n_e}\cdotp {K}_D^T $$

where *K*_*D*_^*T*^ is the thermal distribution coefficient (m^3^ kg^−1^), *ρ*_*b*_ is the bulk density (kg m^−3^), and *n*_*e*_ is the effective porosity of the aquifer. The value of the thermal distribution coefficient *K*_*D*_^*T*^ (quotient between specific heat capacity of the solid and volumetric heat capacity of water) was subject to calibration. The thermal diffusion coefficient (*D*_*m*_^*T*^) was set to 2.9 × 10^−6^ m s^−1^ and was held fixed during modeling.

Average travel times of the recharged water from the canal into the aquifer (Fig. 1 d) were estimated based on the semi-analytical forward particle tracking scheme (based on the Pollock method (Pollock 2012)) that is implemented in ORTI3D (Verardo et al. 2017). These estimated average travel times were additionally compared with temperature breakthrough times and advection-dispersion times of conservative tracer to prove the consistency of the flow model.

#### Multispecies reactive transport model

In this study, we have set up a reactive network to simulate redox reactions as the basis for the degradation of organic micropollutants. To describe the degradation rate (*r*) of oxygen and nitrate when oxidizing dissolved or particulate organic matter, we used common Monod kinetics (Lu et al. 1999; Greskowiak et al. 2006) for a substance *A*:

2 $$ r={k}_{\mathit{\max},A}\cdotp Y\cdotp \frac{C_A}{K_{m.A}+{C}_A}\cdotp \frac{K_{inh,H}}{K_{inh,H}+{C}_H}\cdotp {f}_{TMP} $$

where *k*_*max*_ is the maximum consumption rate constant (mol L^−1^ s^−1^), *Y* is the stoichiometric factor (-), *C*_*A*_ is the concentration of *A* (mol L^−1^), *K*_*m*,*A*_ is the Monod half-saturation constant (mol L^−1^) of the considered species, *C*_*H*_ is the concentration (mol L^−1^), and *K*_*inh*,*H*_ is the inhibition coefficient (mol L^−1^) for an inhibiting species *H*, and *f*_*TMP*_ is a temperature factor. In groundwater environments, the biodegradation rates are controlled, e.g., by terminal electron acceptor processes (TEAPs). TEAPs were approximated as occurring sequentially, with the highest energy-yielding electron acceptors (oxygen) consumed before those that yield less energy (nitrate), e.g., the degradation rate of nitrate is inhibited by the presence of oxygen. And for the rate of oxygen, the inhibition term is set to 1 because no TEAPs inhibit the rate of oxygen. Only when oxygen is depleted to a level that ${C}_{O_2}\ll {K}_{inh,{O}_2}$ the inhibition term tends to 1 and predominantly denitrification occurs. If oxygen is depleted to less than 1.6 × 10^−5^ mol L^−1^ in groundwater, suboxic to anaerobic conditions result, whereas subsequent redox zones overlap (McMahon and Chapelle 2008; Canfield and Thamdrup 2009). The stoichiometric factor for the reaction of oxygen or nitrate with the organic matter was set to − 1 for degradation of oxygen, and − 0.8 for NO_3_^−^, according to the formulation of the redox reactions of Prommer and Stuyfzand (2005) and Henzler et al. (2016), whereby organic carbon species are represented by CH_2_O. For using Monod kinetics, constant enzyme activity in a flow equilibrium is a condition. An additional Monod term to account for limited availability of electron donors (organic carbon species) was not included in Eq. 2, assuming that bioavailable organic matter was unlimited during the simulated time, applying a Monod term to be close to 1 (Munz et al. 2019).

The degradation of organic micropollutants was approximated as 1st order degradation including a Monod term to account for the oxygen level or inhibition terms to simulate the inhibition of anoxic pathway degradation due to the presence of the electron acceptors oxygen and nitrate:

3 $$ {r}_{OMP, oxic}=-{\lambda}_{\mathit{\max}, OMP}\cdotp {C}_{OMP}\cdotp \frac{C_{O_2}}{K_{O_2}+{C}_{O_2}}\cdotp {f}_{TMP} $$

4 $$ {r}_{OMP, anoxic}=-{\lambda}_{\max, OMP}\cdot {C}_{OMP}\cdot \frac{K_{inh,{O}_2}}{K_{inh,{O}_2}+{C}_{O_2}}\cdot \frac{K_{inh,{NO}_3^{-}}}{K_{inh,{NO}_3^{-}}+{C}_{N{O}_3^{-}}}\cdot {f}_{TMP} $$

where *λ*_*max*_ is the maximum degradation rate constant of the OMPs. The conditions for degradations and inhibition of specific degradation processes were defined according to the literature in a way that the degradation of phenazone, diclofenac, and valsartan occurs under oxic conditions (Eq. 3) and the degradation of carbamazepine and sulfamethoxazole (Eq. 4) occurs under low concentrations of oxygen and nitrate (Greskowiak et al. 2006; Wiese et al. 2011; Munz et al. 2019). Preliminary model simulations showed that iron and manganese concentrations in reactive modeling can be omitted because the absence of oxygen and nitrate ensure these anaerobic conditions, and the corresponding iron and manganese concentration did not entirely deplete at our transect. This leads to a computational advantage because no more parameters have to be considered than in Eqs. 3 and 4 and calibrated, and also deviations from iron or manganese simulations are not transferred to OMP modeling.

The temperature factor in Eqs. 2–4 represents a normalized empirical function for the dependence of the degradation rate on temperature *T* (°C) which was defined as:

5 $$ {f}_{TMP}=\frac{\mathit{\exp}\left[\beta \cdotp T\left(1-0.5\cdotp \frac{T}{T_{opt}}\right)\right]-1\ }{\mathit{\exp}\left[0.5\cdotp \beta \cdotp {T}_{opt}\right]-1} $$

where *β* is a fitting parameter (°C^−1^) and *T*_*opt*_ is the optimal temperature, at which the highest microorganism activity occurs. *T*_*opt*_ was set to 35 °C (Greskowiak et al. 2006; Diem et al. 2013; Margot et al. 2013; Sadef et al. 2014).

We adapted this empirical function (Eq. 5) from the temperature factor proposed by O’Connell (1990) and Kirschbaum (1995) and other reactive transport simulations of OMPs (e.g., Greskowiak et al. 2006; Sharma et al. 2012; Henzler et al. 2016). The above mentioned modeling studies have applied an exponential empirical function ($\mathit{\exp}\left[\alpha +\beta \cdotp T\left(1-0.5\frac{T}{T_{opt}}\right)\right]$), depending on the two fitting parameters *α* and *β*, in order to more accurately simulate degradation rates at lower temperatures in comparison with the commonly used Arrhenius equation. We modified this empirical function, because mathematically, *α* do not depend on *T* and can be separated from the empirical function, i.e., *exp*^*α*^ is effectively adjusted merely the maximum rate constant. Initially *α* determines the absolute rate of the degradation process and only *β* in conjunction with *T*_*opt*_, its temperature dependence (O’Connell 1990; Kirschbaum 1995); e.g., the absolute rate of the degradation is given by *k*_*max*_ in Eqs. 2–5. Additionally, a normalization was implemented assuming that *T*_*min*_ is equal to zero and that *T*_*max*_ *= T*_*opt*_ in the bank filtration system. Due to the normalization, the values of *f*_*TMP*_ range between zero and 1. Therefore, *f*_*TMP*_ can only have a limiting impact on the maximum rate constant, e.g., to represent the inhibiting role of low temperatures on microorganism activity leading to decreasing reaction rates (Burke et al. 2014). If the current temperature is the same as the optimal temperature, then the factor will be 1 and the degradation rate is determined by the maximum rate constant, multiplied by Monod and inhibition terms. Temperature values higher than *T*_*opt*_ lead to a decrease of the temperature factor in Eq. 5, due to less enzyme activity for some microorganisms such as mesophilic and psychrophilic bacteria by higher temperatures (Moran and Hickey 1997; Berk 2009). Finally, this normalization should provide better comparability between experimentally derived and modeling-based degradation rates. In this study, we followed the approach that lower temperatures reduce degradation rates and thus oxygen consumption, and temperatures above 11 °C lead to a degradation rate which is close to the true maximum rate constant.

The bulk densities of the porous media have been calculated based on effective porosities and densities of the solids and ranged from 1080 kg m^−3^ (canal bottom) to 2490 kg m^−3^ (glacial till). The longitudinal dispersivities were set to 0.5–1 m. In this study, sorption was not applied for the solute transport modeling because it led to larger residence times and stronger damping of concentrations than the measurements let expect. Furthermore, the considered OMPs are known for low sorption behavior (Greskowiak et al. 2006; Wiese et al. 2011; Henzler et al. 2014; Munz et al. 2019).

Measurements of the time-dependent concentration from surface water and from the ambient groundwater (Table 1) were taken as values for a Dirichlet boundary at the canal bottom (SFW in Fig. 1 c) and at the left-hand side of the 2nd aquifer (GW in Fig. 1 c), respectively. If measured concentrations were below the limit of detection (LOD), they were set as half of the LOD for the transient model boundaries.

**Table 1:** Measured range of surface water and ambient groundwater concentrations used for the model boundaries

Water component	Surface water Min – Max [mol L^−1^]	Ambient groundwater Min – Max [mol L^−1^]
O_2_	1.8 × 10^−4^ – 4.5 × 10^−4^	< LOD – 1.9 × 10^−6^
NO_3_^−^	5.0 × 10^−5 ^– 1.5 × 10^−4^	< LOD – 1.9 × 10^−5^
Cl^−^	1.3 × 10^−3^ – 2.2 × 10^−3^	1.2 × 10^−3^ – 1.4 × 10^−3^
Acesulfame	1.2 × 10^−9^ – 3.3 × 10^−9^	5.0 × 10^−9^ – 8.3 × 10^−9^
Phenazone	1.6 × 10^−10 ^– 6.6 × 10^−10^	4.3 × 10^−9^ – 5.8 × 10^−9^
Diclofenac	< LOD – 1.4 × 10^−9^	< LOD
Sulfamethoxazole	9.5 × 10^−11^ – 3.4 × 10^−10^	< LOD
Carbamazepine	3.4 × 10^−10 ^– 1.3 × 10^−9^	< LOD
Valsartan	2.0 × 10^−10 ^– 4.5 × 10^−9^	< LOD

#### Model platform and calibration procedure

Groundwater flow modeling was set up with MODFLOW (Harbaugh et al. 2000) within the user interface ORTI3D (Verardo et al. 2017). ORTI3D (*open reactive transport interface*) is a freely accessible program for modeling groundwater flow, transport, as well as conservative and reactive processes. It is a Python-based user interface, e.g., MODFLOW, MT3DMS, and PHT3D. The transient heat and reactive transport modeling was set up with PHT3D (Prommer and Post 2010), a program coupling the transport simulator MT3DMS (Zheng and Wang 1999) with the hydrogeochemistry simulator PHREEQC-2 (Parkhust and Appelo 1999).

To accurately reproduce the flow and transport conditions, as well as the thermal groundwater conditions at the bank filtration site, the model was manually calibrated concerning horizontal hydraulic conductivities and anisotropy ratio to increase the correlation and to reduce the root mean square error (RMSE) between the measured and simulated hydraulic heads as well as chloride and acesulfame concentrations. Additionally, the thermal distribution coefficient was also manually calibrated to achieve a lower RMSE between the measured and simulated temperatures. Based on the calibrated water flow and heat transport model, the maximum rate constants of the redox parameters and micropollutants, Monod saturation constants, inhibition constants, and the parameter *β* of the temperature factor were calibrated in order to increase correlation and reduce the RMSE between measured and simulated concentrations.

To identify the impact of temperature on reactive transport, a statistical analysis, calculating *p*-values by a Wilcoxon rank sum test, was carried out. Therefore, concentrations, based on reactive modeling with implemented temperature factor or without temperature factor, were compared for time steps at which the measurements were taken and when temperatures were below 11 °C (*n* = 16). The temperature factor was omitted in the kinetic reactions of each solute (Eqs. 2, 3, 4), but the OMP reactions still depend on the temperature-dependent concentration of redox parameters (Eq. 2) and thus indirectly on temperature. For *p*-values smaller than 0.01, the temperature has a significant impact on solute consumption and degradation (cp. 4.3).

## Results and discussion

### Water flow, conservative tracers, and heat transport

**Fig. 2:**
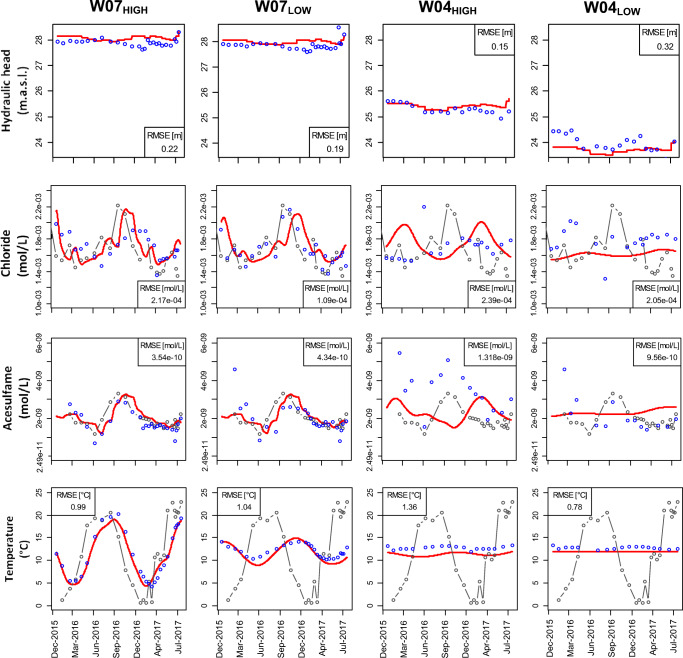
Plot of simulated (red lines) and observed (blue circles) hydraulic heads, chloride concentrations, acesulfame concentrations, and temperature at W07_HIGH_, W07_LOW_, W04_HIGH_, and W04_LOW_. RMSE values are determined for each observation point. Grey-dotted lines represent the observed canal concentrations and temperatures

The calibrated transient 2D water flow and heat transport clearly represent the natural occurring thermal groundwater conditions at the studied bank filtration system. Values for the calibrated hydraulic conductivities are presented in Table 2. In our study, only a slight adjustment of hydraulic conductivities was desired, as being at hand from a 3D model already, and with our calibrated hydraulic heads, we were able to get mostly better agreement than Wang et al. (2020). The simulation of the hydraulic heads justifies our simplifying assumptions with respect to the model boundaries and the aquifer’s hydrogeology (Fig. 2). The short-term changes in the pumping rate could not be formulated as a boundary condition due to such data not being available. Therefore, the model cannot be expected to reproduce the observed short-term fluctuations of the hydraulic head levels at the W04_LOW_.

**Table 2:** Calibrated horizontal and vertical hydraulic conductivities for the transect as in Figure 1 c

Zones	Horizontal hydraulic conductivity [m s^−1^]	Anisotropy ratio [-]
Canal bed	1.6 × 10^−6^	5
Silt lens	2.0 × 10^−6^	10
First aquifer	1.2 × 10^−4^	2.3
Semipermeable glacial till	4.1 × 10^−6^	2.5
Second aquifer - upper part	2.3 × 10^−5^	2.5
Second aquifer - lower part	5.8 × 10^−6^	5.3

The simulated concentrations of chloride and acesulfame highlight the quality of model calibration especially in W07_HIGH_ and W07_LOW_ (Fig. 2). A further improvement in model calibration at W07_HIGH_ (e.g., timing of maximum tracer concentration) would imply a less accurate fit in W07_LOW._ This effect could be attributed to any unknown, local heterogeneity of the first groundwater aquifer that is represented by homogeneous hydraulic parameters in the model only. Acesulfame concentrations in W04_HIGH_ are higher than in W07_HIGH_. This suggests that concentrations in W04_HIGH_ and W07 actually do not share exactly the same flow path or are the result of several flow paths mixing. The missing temporal variation in simulated concentrations at W04_LOW_ might be caused by the missing dynamics of hydraulic heads in the deep aquifer. However, these deviations are not an index for a systematic mismatch.

**Fig. 3:**
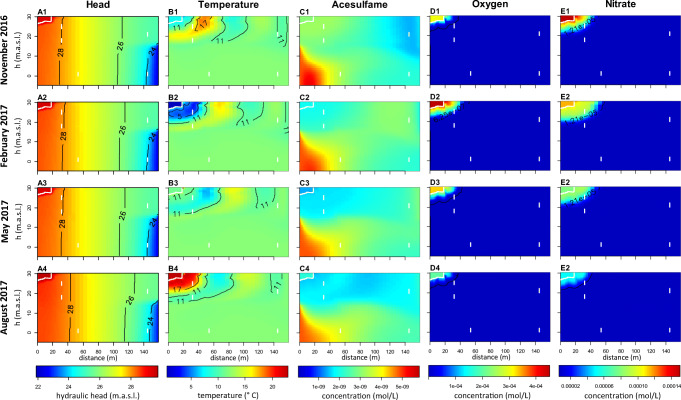
2D plots for the study site transect for four dates in 2016 and 2017 which represent seasonal variation over the year. For hydraulic heads, water temperatures, acesulfame, oxygen, and nitrate. White lines stand for the observation wells and the canal boundary, black line represents certain hydraulic heads and temperatures in A and B; in D-E, the LOD concentration of each species

The spatial distributions of hydraulic heads along the model transect is shown in Fig. 3. The simulated decrease in hydraulic heads from the surface water (${\overline{h}}_{SFW}$ = 29.5 m) to the pumping well (${\overline{h}}_{PW}$ = 22.5 m) reproduced well the natural conditions at the study site. The hydraulic gradient between the canal and the pumping well situated in the second aquifer was determined as 0.04 m m^−1^. The resulting flow directions in both aquifers were dominantly horizontal from the left-hand side towards the right-hand side of the transect with a slight downward component that increased towards the very right-hand side model boundary representing the pumping well (Fig. 1 d). The flow lines indicate that the upper aquifer (W07_HIGH_, W07_LOW_, and W04_HIGH_) is dominated by surface water infiltration from the canal, whereas the lower aquifer (W03_LOW_ and W04_LOW_) is mainly receiving an ambient groundwater inflow. For W04_LOW_, there is some influence from bank filtration water at the northern shore (outside the model transect) that has moved down and south (by the pumping), and possibly also some contribution via mixing from water having passed the upper aquifer close to the pumping well (Fig. 1 d). Overall, concentrations in W03 and W04_LOW_ must not be seen as being downstream of flowlines through W07 (or W04_HIGH_) due to this vertical displacement and also by their horizontal offset.

The residence times in the first more permeable aquifer were shorter than in the second aquifer (Fig. 1 d). After 150 d, dissolved solutes arrived at the PW boundary (Fig. 1 d). The residence times of our study are consistent when comparing conservative tracers and furthermore correspond to estimated residence times of existing studies of this field site (Munz et al. 2019; Wang et al. 2020). Simulations of Wang et al. (2020) highlighted that the quickest flow path reached the pumping wells after 100 d and that about 20% of the particle tracks from the canal reached the pumping wells after 150 d. The spatial distributions of hydraulic heads along the model transect (Fig. 3 A1, A2, A3, A4), the resulting flow field, and the subsurface residence times did not substantially vary over time.

The simulated temperatures accurately represent the averaged observed temperatures as well as the temporal characteristics of the temperature signal (Fig. 2). The high variability from 1.8 to 19.3 °C and the timing of the measured temperature signal for W07_HIGH_ are well captured by the model. Along the flow direction, the simulated temperatures were reduced in amplitude and shifted in time in respect to canal temperatures.

The spatial distribution of temperature along the model transect over time is shown in Fig. 3 B1, B2, B3, B4. Water temperature values close to the canal represented the seasonal dynamics of the surface water temperature with the lowest temperature values in February 2017 (Fig. 3 B2) and the highest ones in August 2017 (Fig. 3 B4). These temperature signals were transported into the first aquifer. With increasing distance from the canal, the temperature amplitude in the first aquifer (deviation of temperature from the seasonal average temperature of about 3 °C) continuously decreased. At a distance of 140 m from the canal, the simulated amplitude was lower than 1 °C (Fig. 3). In the second aquifer, the temperature was almost constant in space and time ($\overline{T}$ = 12 °C).

The most sensitive parameters affecting groundwater temperature distribution are hydraulic conductivity and thermal distribution coefficient ${K}_D^T$. During the calibration, ${K}_D^T$ was found to be sensitive to the temperature amplitude in the upper and lower aquifers. The optimized thermal distribution coefficients were 3.4 × 10^−4^ m^3^ kg^−1^ (*R* = 3.3) for the first aquifer, 6.7 × 10^−4^ m^3^ kg^−1^ (*R* = 9.6) for a zone around the fractured aquitard, and 6.0 × 10^−4^ m^3^ kg^−1^ (*R* = 5.1) for the second aquifer. Thus, the calibrated *R*-value of the first aquifer is in the range found by Engelhardt et al. (2013a) for similar quaternary sediments with grain sizes varying between fine sand and fine gravel (2.4 < *R* < 3.6). The calibrated values caused a strong dampening of the temperature signal in the lower aquifer and yield the almost constant temperatures as observed. The calibrated *K*_*D*_^*T*^ for the second aquifer is 6.0 × 10^−4^ m^3^ kg^−1^ and thus about two times higher than our value of the first aquifer. Notwithstanding, it still is in the plausible range of values calculated from literature based on the specific heat capacity of the solid of comparable sediment types (Park et al. 2015; Epting et al. 2013; Stonestrom and Blasch 2003) and the volumetric heat capacity of water. The resulting *K*_*D*_^*T*^ ranged between 5.1 × 10^−4^ m^3^ kg^−1^ for silty sand (Park et al. 2015), 6.19 × 10^−4^ m^3^ kg^−1^ for Tottori sand (Stonestrom and Blasch 2003), and 6.83 × 10^−4^ m^3^ kg^−1^ for gravel (Epting et al. 2013). Following Eq. 1, this would lead to *R* values up to 5.6 (*ρ*_*b*_ = 1560 kg m^−3^; *n*_*e*_ = 0.23) for sand and gravel aquifers of alluvial and glacial origin. Our study site contains heterogeneous geological conditions (Wang et al. 2020) including silty sand and glacial till which may include gravel components. Notably, at our site, the watercourse is an artificial canal dug into glacial deposits, which could well make a difference to natural watercourses and their riverbeds. Therefore, it is plausible that the values of the thermal retardation factor at our site can be truly larger than *R* adopted at other nearby bank filtration sites, e.g., at lake Wannsee in Berlin (Greskowiak et al. 2006; Massmann et al. 2009).

### Reactive transport modelling—oxygen and nitrate

**Fig. 4:**
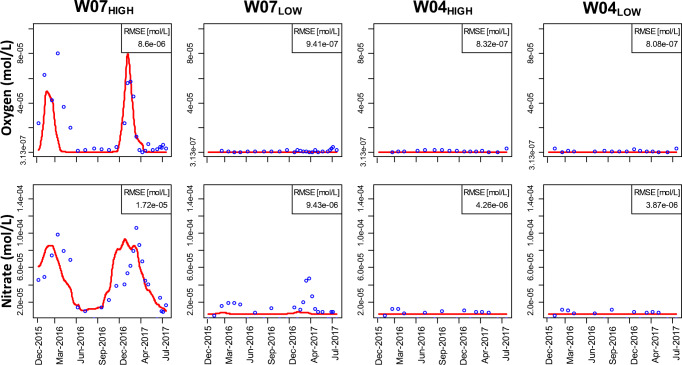
Plot of simulated (red lines) and observed (blue circles) oxygen and nitrate concentrations over time at W07_HIGH_, W07_LOW_, W04_HIGH_, and W04_LOW_. RMSE values are determined for each observation point

The calibrated reactive transport model was able to capture the spatial and temporal variations in observed oxygen and nitrate concentrations (Fig. 4). For the temperature factor, the optimal temperature was held fixed as T_opt_ = 35 °C and the parameter *β* was determined as − 0.25 °C^−1^ and will be discussed under the “Temperature dependent reaction kinetics” section. The calibrated maximum rate constants and Monod saturation and inhibition constants are listed in Table 3. Substantial variations in oxygen were simulated for W07_HIGH_. The highest deviations between simulated and observed oxygen concentrations for W07_HIGH_ occurred in winter 2016 where the observed breakthrough of oxygen concentrations was underestimated by the model (Fig. 4). Simulated concentrations of oxygen in the observation points W03 and W04 were close to the LOD over the entire simulation period.

**Table 3:** Calibrated reactive transport parameters for redox-sensitive species and organic micropollutants

Solutes	Maximum rate constant *k*_*max*_^*1*^, *λ*_*max*_^*2*^	Monod saturation constant *K*_*m*_ [mol L^−1^]	Monod inhibition constant *K*_*inh*_ [mol L^−1^]
*Oxygen*^*1*^	4.72 × 10^−10^ [mol L^−1^ s^−1^]	1.0 × 10^−5^	-
*Nitrate*^*1*^	3.70 × 10^−11^ [mol L^−1^ s^−1^]	4.0 × 10^−5^	O_2_: 2.0 × 10^−5^
*Phenazone*^*2*^	6.60 × 10^−6^ [s^−1^]	4.0 × 10^−4^	-
*Diclofenac*^*2*^	2.00 × 10^−6^ [s^−1^]	1.0 × 10^−6^	-
*Valsartan*^*2*^	7.60 × 10^−6^ [s^−1^]	1.0 × 10^−4^	-
*Sulfamethoxazole*^*2*^	1.30 × 10^−6^ [s^−1^]	-	O_2_: 1.0 × 10^−5^/NO_3_^−^: 2.0 × 10^−5^
*Carbamazepine*^*2*^	1.30 × 10^−6^ [s^−1^]	-	O_2_: 5.0 × 10^−7^/NO_3_^−^: 8.0 × 10^−6^

Substantial variations in nitrate concentrations were simulated for W07_HIGH_ (Fig. 4). The breakthrough of nitrate was slightly underestimated in winter 2016 and slightly overestimated by the model in winter 2017 in comparison with the observations. For W07_LOW_, relevant nitrate concentrations were observed for some periods of time. Since in the simulation oxygen in W07_LOW_ is almost depleted, nitrate degradation is not inhibited and nitrate is reduced to large degree. By that, a small deviation in simulated oxygen concentrations can cause a partial underestimation of observed nitrate concentrations in W07_LOW_. In other words, in spring time, W07_LOW_ seems to be located just in the transition zone from oxygen to nitrate reduction. This sensitive positioning should not mislead the calibration effort to implement a stronger inhibition even by very low oxygen concentrations or a lower maximum rate constant since this would worsen the consistency in W07_HIGH_ unjustified. Thus, our model results and parameters show the best-balanced calibration result for W07_HIGH & LOW_ together.

The simulated oxygen and nitrate distribution captured the measured values in 2017 well but underestimated them between January and May 2016. Therefore, we focus the interpretation on the results of November 2016 until August 2017. Generally, high oxygen concentrations from the canal were transported into the first aquifer over the whole simulation time (Fig. 3 D). The oxygen concentrations in the canal were highest in February 2017 and lowest in August 2017. In February 2017, the concentration plume extended about 40 m horizontally from the canal into the first aquifer and up to 10 m in the vertical direction (mean residence time *t*_*res*_ = 20 d, Fig. 3 D2). In August 2017, the penetration of oxygen into the first aquifer has weakened and only reached to 30 m in horizontal and 5 m in the vertical direction (*t*_*res*_ = 10 d, Fig. 3 D4). For the rest of the model domain, the simulated concentrations were close to half the LOD concentration.

The distribution of nitrate concentrations in the aquifer show a similar pattern as oxygen (Fig. 4), just extending somewhat further downstream as to be expected. In February 2017, the concentration plume extended about 60 m from the canal horizontally into the first aquifer and up to 15 m in the vertical direction (*t*_*res*_ = 35 d, Fig. 3 E2). In August 2017, the penetration of nitrate into the first aquifer only reached to 40 m in horizontal and up to 10 m in the vertical direction (*t*_*res*_ = 20 d, Fig. 3 E4). For the rest of the model domain, the simulated concentrations were close to half the LOD concentration.

The occurrence and extent of oxygen and nitrate strongly varied in space and time in dependence of the seasonal variation in temperature of the recharge water (Fig. 3). During winter (water temperatures below 8 °C), the microbial activity was generally lower, and the dissolved oxygen and nitrate substantially infiltrated into the aquifer. During summer (water temperatures above 11 °C), the microbial activity was generally larger, and the dissolved oxygen became depleted within the first meters of the aquifer followed by the nitrate-reducing zone. The resulting zonation of redox parameters on bank filtration sites has been intensively described in the last years (for example Massmann et al. 2009; Sharma et al. 2012; Henzler et al. 2016). All of them pointed out that besides the subsurface residence time, also the seasonal temperature dynamics strongly influenced the zonation of redox parameters.

The determined maximum rate constant of oxygen (4.72 × 10^−10^ mol L^−1^ s^−1^) is in the same order of magnitude as found in previous studies (Greskowiak et al. 2006; Henzler et al. 2016). Denitrification is described in the literature as a process, which can occur under various oxygen thresholds of about 3.2 × 10^−6^ mol L^−1^ s^−1^ to 3.2 × 10^−5^ mol L^−1^ s^−1^ representing dominantly anaerobic conditions (McMahon and Chapelle 2008; Rivett et al. 2008). We found that nitrate degradation started to occur a bit earlier, that is, if oxygen is depleted to less than 4 × 10^−5^ mol L^−1^ in the groundwater. A slight inhibition of nitrate consumption through oxygen was implemented (Table 3), which lead to an inhibition by oxygen concentrations starting above 4.0 × 10^−5^ mol L^−1^. Therefore, a good agreement in W07_HIGH_ was achieved where relevant nitrate concentrations break through. In our study, the estimated *k*_*NO*__*3*_*-* value is nearly 1 magnitude lower as found by Henzler et al. (2016) but is in the same order of magnitude as found by Greskowiak et al. (2006) (see Table 3).

### Reactive transport modeling – micropollutants

#### Degradation under oxic conditions

**Fig. 5:**
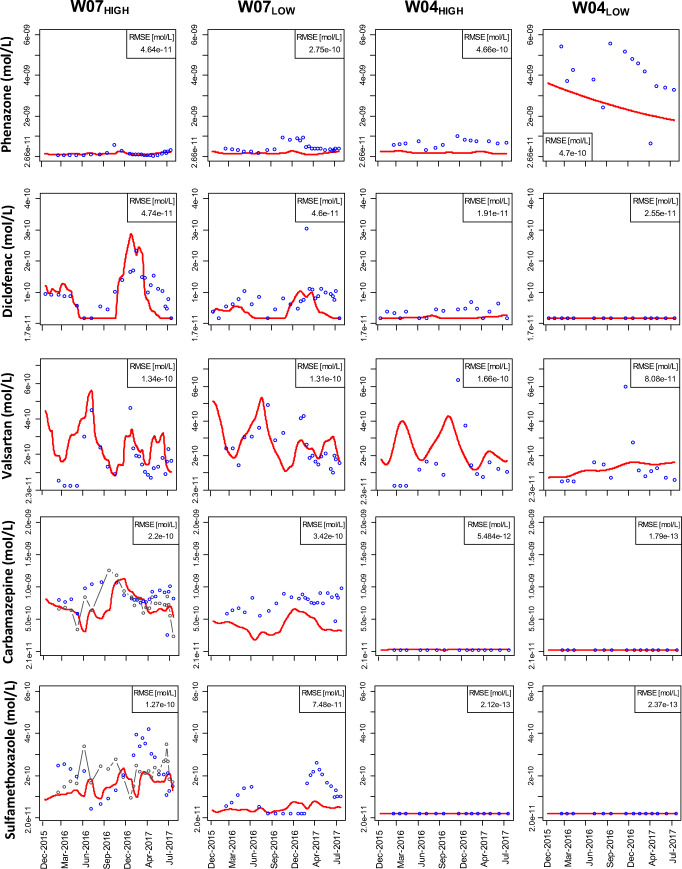
Breakthrough curves of simulated (red lines) and observed (blue circles) concentrations of phenazone, diclofenac, valsartan, carbamazepine, and sulfamethoxazole at W07_HIGH_, W07_LOW_, W04_HIGH_, and W04_LOW_. the observed canal concentrations of carbamazepine or sulfamethoxazole are indicated by grey-dotted lines. RMSE values are determined for each observation point

The calibrated temperature-dependent reactive transport model was able to capture the spatial and temporal variations in observed phenazone, diclofenac, and valsartan concentrations (Fig. 5) on average. The effect of varying temperatures (temperature factor) on the degradation rate is intensively discussed in the “Temperature-dependent reaction kinetics” section. The calibrated reactive transport parameters for the organic micropollutants are presented in Table 3.

**Fig. 6:**
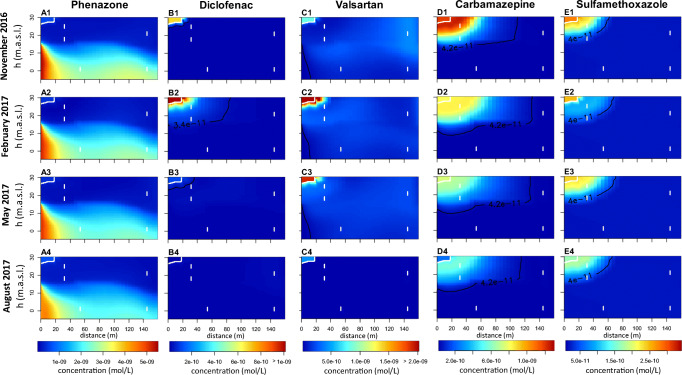
2D plots for the study site transect for four dates in 2016 and 2017 which represent seasonal variation over the year. For phenazone, diclofenac, valsartan, carbamazepine, and sulfamethoxazole. White lines stand for the observation wells and the canal boundary; black line represents the LOD-concentration of each species

The spatial distribution of simulated OMP concentrations along the model transect over time is shown in Fig. 6. The phenazone concentrations in the canal were highest in August 2017 and lowest in May. Phenazone was transported only a few meters into the first aquifer (*t*_*res*_ < 10 d). The highest concentrations occurred within the entire second aquifer (Fig. 5 and Fig. 6 A1, A2, A3, A4), originating from a latent subsurface flux, indicating a higher input in the past, which was already assumed by Reddersen et al. (2002).

The diclofenac concentrations in the canal were highest in February 2017 and lowest in August 2017. In February 2017, the diclofenac concentration plume extended up to 80 m from the canal horizontally into the first aquifer and up to 25 m in the vertical direction (*t*_*res*_ = 60 d, Fig. 6 B2). A strong decrease took place within *t*_*res*_ = 20 d. In August 2017, penetration of diclofenac into the first aquifer approaches zero (Fig. 6 B4). For the rest of the model domain, the simulated concentrations were close to half the LOD concentration and match the observed values very well (Fig. 5).

The valsartan concentrations in the canal were highest in February 2017 and lowest in August 2017. Except for August 2017, over the entire simulation period, valsartan occurred in the entire first and second aquifer up the right model boundary (Fig. 6 C1, C2, C3, C4). Despite the high degradation rate constant, valsartan was still detected within the entire aquifer due to the high source concentrations and the strong dependence of the degradation rate on the presence of oxygen. Thus, especially in winter when temperatures are low, substantial valsartan concentrations penetrated into the aquifer and were further transported through the anoxic zones without further degradation and thus may reach this pumping well.

During summer (water temperatures above 11 °C), it can be assumed that the microbial activity was generally higher because less phenazone, diclofenac, and valsartan were present in the first aquifer than in winter (water temperatures below 11 °C). Additionally, low concentrations in surface water during high water temperatures and low oxygen concentrations exist. The absolute removal was higher in winter with higher oxygen concentrations. If the temperature was below 11 °C, oxygen concentration was high enough (up to 2.0 × 10^−4^ mol L^−1^) for diclofenac degradation and the diclofenac plume concentration decreases below the LOD in the horizontal direction after *t*_*res*_ = 60 d (Fig. 3 B2 and Fig. 6 B2). The calibrated Monod half-saturation constants for phenazone, diclofenac, and valsartan (Table 3) represent a strong dependency of the degradation rate on oxygen concentrations. The phenazone and valsartan degradation is more strongly limited by the presence of oxygen than diclofenac (cp. Fig. 4). How important the presence of oxygen is for phenazone and valsartan degradation has previously been identified (Greskowiak et al. 2006; Massmann et al. 2009; Burke et al. 2018; Munz et al. 2019). Due to the high oxygen dependency, phenazone, diclofenac, and valsartan were not degraded in the second aquifer. Anoxic conditions inhibit the degradation of these OMPs (Greskowiak et al. 2006; Wiese et al. 2011; Munz et al. 2019). Therefore, higher concentrations of phenazone are present in the groundwater inflow in the deeper aquifer, given its anoxic conditions and temperatures around 11–12 °C. Due to that, concentrations did not exceed a health threshold of 0.1 μg L^−1^ (UBA 2017) at the nearby pumping well of the adjacent waterworks.

Generally, the seasonal variations in the fate of organic micropollutants degrading under oxic conditions in the aquifer were shown to be controlled by their concentration in the surface water, the overall maximum rate constant dependent on the presence/absence of oxygen, and partly by the temperature of the recharge water. The calibrated degradation rates in combination with the low source concentration of phenazone and diclofenac in the canal are sufficient for a complete removal of these substances at the nearshore aquifer throughout the entire observation period.

#### Degradation in an anoxic environment

The calibrated reactive transport model was able to capture the spatial and temporal variations in observed sulfamethoxazole and carbamazepine concentrations (Fig. 5) on average sufficiently well. The observed sulfamethoxazole and carbamazepine concentrations were partially higher at W07_HIGH_ than in the canal, while the measured canal concentrations are used as input concentration boundary condition and are additionally shown in Fig. 5 for carbamazepine and sulfamethoxazole. The time-shift of the simulated concentrations compared with the measured canal concentration clearly shows the expected transience in time of the input signal towards W07. But for most times of the year, the measured groundwater concentrations exceed those of the surface water. These deviations are the result of limitations in the experimental data set, which is peak concentrations in the SFW missed by the sampling scheme, which clearly cannot be resolved by the model. Further deviations between observed and simulated concentrations occurred mainly for sulfamethoxazole, especially in W07_LOW_ in periods when oxygen and nitrate concentrations were not matched exactly (cp. Figs. 4 and 5).

In our study, we determined the maximum rate constant for carbamazepine and sulfamethoxazole for both with 1.3 × 10^−6^ s^−1^ with regard to Monod kinetic (Table 3). The calibrated Monod half-saturation constants for carbamazepine and sulfamethoxazole represent the strong inhibition by high oxygen and nitrate concentrations but the carbamazepine degradation is more strongly reduced than sulfamethoxazole (Table 3). Therefore, we point out that carbamazepine degradation only occurred under fully anaerobic conditions, which is supported by observed concentrations.

The temporal and spatial patterns of the carbamazepine and sulfamethoxazole concentrations were comparable to each other. The concentration of both OMPs was highest in November 2016 and lowest in August 2017. The penetration depth into the first aquifer was highest in February and May 2017 with travel distance up to 120 m (*t*_*res*_ = 95) and 70 m (*t*_*res*_ = 60 d) in horizontal direction for carbamazepine and sulfamethoxazole, respectively (Fig. 6 D and E). During the whole simulation period, degradation below the LOD was occurring in less than *t*_*res*_ = 4 months for carbamazepine and in about *t*_*res*_ = 2 months for sulfamethoxazole.

Taking into account the nitrate concentration for simulations based on our measurements which show an overlap between the oxic and denitrification zone, also high nitrate concentrations can occur seasonally with high oxygen concentrations. This lead to a suboxic transition zone, which could provide inhibiting condition to anaerobic bacteria activity. Studies pointed out that sulfamethoxazole and carbamazepine can degrade under anaerobic conditions, where iron, manganese, or sulfate reduction take part; thereby sulfamethoxazole degradation can also partly occur under suboxic or denitrifying conditions (Grünheid et al. 2005; Stuyfzand et al. 2007; Wiese et al. 2011). Therefore, sulfamethoxazole is less strongly inhibited by nitrate than carbamazepine which is reflected by a calibrated lower inhibition constant. Based on these results, our model approach that carbamazepine and sulfamethoxazole mainly degraded if oxygen and nitrate are nearly depleted in the aquifer provides anaerobic conditions that are most suitable for these both OMPs.

Earlier studies pointed out that carbamazepine is nearly persistent or shows only low removal (Fenz et al. 2005; Wiese et al. 2011; Burke et al. 2018). On opposite, in our study, we determined a complete carbamazepine removal (to below LOD concentration) under anoxic conditions on the longest flow path in the first aquifer (at W04_HIGH_).

Other studies of bank filtration sites, which investigate sulfamethoxazole, determined low to high removal in groundwater and first-order degradation constant which is one order of magnitude lower than in the maximum rate constant in our study (Henzler et al. 2014). For the same study site, the analytically determined degradation rate for carbamazepine and sulfamethoxazole were 4.5 × 10^−7^ s^−1^ and 4.2 × 10^−7^ s^−1^ (Munz et al. 2019), respectively, which is one order of magnitude lower than the *k*_*max*_ in the present study. However, both studies estimated yearly averaged first-order degradation rates which did not include redox and/or temperature dependencies. The implemented Monod terms in our study could lead to a factor of about 1 to 10^−3^ for the observed range in oxygen concentrations and could, therefore, explain the differences between the maximum consumption rate and the maximum degradation rate above. These results highlight that the temporally and spatially varying, highly dynamic redox conditions and temperature changes typically characterizing a BF system should be taken into account for an accurate estimation of actual degradation rates. In this study, the main observed degradation behavior of all OMPs could be simulated.

### Temperature-dependent reaction kinetics

**Fig. 7:**
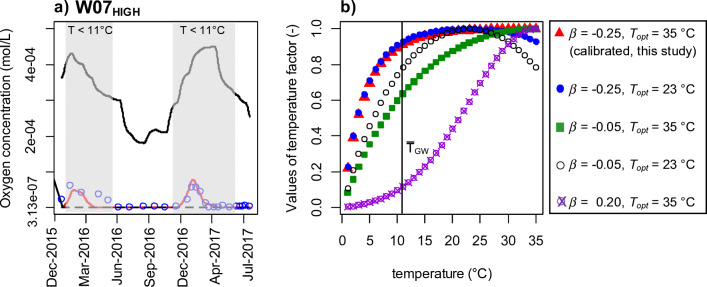
(a) Time-series graph of W07_HIGH_ for oxygen concentrations. Red line, with calibrated temperature factor (*β* = − 0.25, *T*_*opt*_ = 35 °C); black dashed line, without temperature dependence, e.g. the temperature factor was set to unity. Both simulations were carried out with the same *k*_*max*_ and Monod terms. Black solid line shows concentration for non-reactive transport. Blue circles are the observed concentrations. Grey rectangles show the time periods where groundwater temperature was below 11 °C. (b) Value of temperature factor [-] against temperature [°C] with different parameterizations of *β and T*_*opt*_. Black straight line with T̅_GW_ stands for the average groundwater temperature for our transect

The applied and calibrated temperature factor (*β* = − 0.25 °C^−1^, *T*_*opt*_ = 35 °C) was necessary to achieve a good match between measured and simulated oxygen concentrations (Fig. 7 a) and thus also indirectly for OMPs via prevalence of favorable or non-favorable degradation conditions.

The observed oxygen concentrations show a clear difference in oxygen consumption for the low temperature range. For this, the temperature factor was calibrated. The function of the calibrated temperature factor highlights the substantial reduction of the maximum consumption rate below temperatures of about 11 °C (Fig. 7 b). For 5 °C, the temperature factor is 0.5, which means degradation rate at this temperature is decreased by 50%. For *T* = 11 °C, which is close to the mean aquifer temperatures, *f*_*TMP*_ is about 0.9, which means the degradation is just diminished by 10% (Fig. 7 b). This corresponds to the assumption that above a certain temperature threshold, degradation by microorganism is not substantially limited by temperature and that the microbial activity is high enough to provide actual degradation rates close to the maximum rate constant. For temperatures above 11 °C, the temperature factor increases to 1 at *T*_*opt*_ = 35 °C. In contrast, a positive beta, e.g., *β* = 0.2 °C^−1^, leads to the highest variations in the temperature factor by temperatures between 15 and 30 °C and would lead to an overall strong decrease for temperatures below the mean groundwater temperature at our study site (variance in temperature factor only up to 10% for *T* < 11 °C with *β* = 0.2, see Fig. 7 b). The calibrated shape of the curve (Fig. 7 b, *β* = − 0.25 °C^−1^) is the result of the calibration and the measured values behind.

**Fig. 8:**
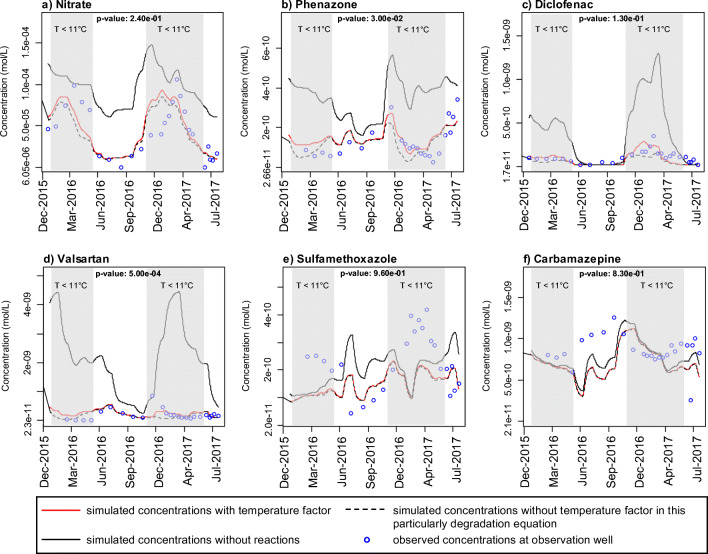
Plot of simulated and observed concentrations over time for W07_HIGH_: (a) nitrate, (b) phenazone, (c) diclofenac, (d) valsartan, (e) sulfamethoxazole, and (f) carbamazepine. Grey background marks the time periods where groundwater temperature was below 11 °C during which *p*-values are determined for applying a temperature function in the particular reaction or not. Note that the red solid and the black dashed lines in subfigures e and f are almost identical.

It has to be stressed that the temperature factor is designed to inhibit degradation at low temperatures. But the most significant impact on degradation is the maximum degradation rate constant, *k*_*max*_. This *k*_*max*_ determines mainly the quantity of solutes which is degraded (Fig. 8). The temperature factor has the function of fitting the curve, so that at lower temperatures, less degradation takes place than at higher temperatures and that in our case there is also a significant difference between lower and medium temperatures.

Additionally, to evaluate the impact of the temperature function on the proposed degradation rates, sensitivity simulations with varying *β*-values and optimal temperatures were carried out (Table 4). These simulations showed that for increasing *β*-values, the optimal maximum rate constant becomes higher and a less good fitting could be achieved due to an excessive straightening of the breakthrough curves. The corresponding results and RMSE for oxygen for different *β*-values are presented in Table 4. For increasing *β*s (*T*_*opt*_ was held fixed as 35 °C), *k*_*max*_ has to be increased to achieve oxygen concentrations in the same order of magnitude as observed. Notably, only one *β* was determined for all kinetic reactions.

**Table 4:** Calibrated maximum rate constant *k*_*max*_ for oxygen concentration for different *β*-values. With considered RMSE for the observation well W07_HIGH_

*β* value [-]	Maximum rate constant *k*_*max*_ [mol L^−1^ s^−1^]	RMSE [mol L^−1^]
− 0.4	2.80 × 10^−10^	3.0 × 10^−5^
− 0.3	4.27 × 10^−10^	1.1 × 10^−5^
**− 0.25**	**4.72** × **10**^−**10**^	**8.6** × **10**^−**6**^
− 0.2	5.28 × 10^−10^	1.1 × 10^−5^
− 0.1	7.40 × 10^−10^	1.3 × 10^−5^
− 0.05	9.95 × 10^−10^	1.3 × 10^−5^
0.05	1.88 × 10^−9^	1.4 × 10^−5^
0.1	2.90 × 10^−9^	1.5 × 10^−5^
0.2	7.90 × 10^−9^	1.6 × 10^−5^
0.3	2.50 × 10^−8^	1.6 × 10^−5^
0.4	9.00 × 10^−8^	1.6 × 10^−5^

The simulation results showed also that the optimal temperature was not a sensitive parameter if the absolute value of *β* was above ± 0.2 (cp. Fig. 7 b). Also Greskowiak et al. (2006) and Kirschbaum (1995) reported that variations of *T*_*opt*_ within the range of the literature values affected the simulation results only marginally. Generally, this simplifies the parameterization of temperature-dependent reaction kinetics; especially with respect to the limited knowledge of the temperature range when microorganism communities in natural aquifers and groundwater will reach their optimum.

The direct temperature dependence due to the applied temperature factor leads to statistically significant differences in oxygen and valsartan compared with simulations without implemented temperature dependence for the solute (*p*-value < 0.01, cp. Figs. 7 a and 8 d). The statistical test was carried out for periods where the measurements were taken and with a temperature below 11 °C (*n* = 16). The temperature factor was omitted in the kinetic reactions of each solute (Eqs. 2, 3, 4) but the OMP reactions still depend on the temperature-dependent redox parameter concentration (Eq. 2) and thus indirectly on temperature. It has to be noted that the observed oxygen concentrations could be also approximated by simulations without the temperature dependence with a smaller rate constant, but this would be accompanied by an overall lower accuracy between observed and simulated values especially at the rising and falling limbs of the oxygen breakthrough curves. Therefore, especially in winter months at lower water temperatures, differences exist due to the reduced consumption or degradation rate by the temperature factor. The concentrations of oxygen, nitrate, and the OMPs degrading under oxic conditions were generally lower if the influence of temperature was not considered in the respective transport equation. The maximum percentage deviation between the simulated concentrations considering the temperature factor and the simulated concentrations without temperature factor in this particular degradation equation at times where the measurements were taken (cp. Fig. 8) was highest for oxygen (98%), followed by valsartan (79%), phenazone (63%), diclofenac (55%), and nitrate (25%). For T > 11 °C, the temperature factor is close to 1 which means that it does not substantially decrease the consumption rate for those times (Fig. 8). For sulfamethoxazole and carbamazepine, the degradation depends strongly on clearly anaerobic conditions which did occur (at least partly) along the transect between canal and W07, with exception from about December–March 2016 and 2017 (Fig. 3 D3 and Fig. 3). That is, for times when temperatures were below 11 °C, oxygen substantially penetrated into the aquifer and inhibited the sulfamethoxazole and carbamazepine degradation (i.e., for those times the non-reactive curve is close to the reactive curve, Fig. 8 e and f) and finally also the temperature function did not have a direct effect on the degradation rates of sulfamethoxazole and carbamazepine. The oxygen availability, however, leads to an indirect temperature dependence on the fate of substances degrading in anoxic environments.

## Conclusions

In our study, a 2D model was created to simulate oxygen-, nitrate-, and temperature-dependent fate and attenuation of six OMPs at a bank filtration site in Germany. The model was calibrated to constrain estimates of the most sensitive model parameters. Simulations of a vertical cross section along a flowline matched the observations over the simulation time of one and a half years. Observation points that are not directly located on the flow path complicate the calibration but the results matched the average. The groundwater hydrogeochemistry along the studied transect substantially varies in space and time and is driven by the input of thermal energy, oxygen, and nitrate from the surface water. Monod kinetic parameters with maximum rate constants were determined for the OMPs phenazone, diclofenac, valsartan, carbamazepine, and sulfamethoxazole for this bank filtration site. Preferential biodegradation of phenazone (*k*_*max*_ = 6.6 × 10^−6^ mol L^−1^ s^−1^), diclofenac (2.0 × 10^−6^ mol L^−1^ s^−1^), and valsartan (7.6 × 10^−6^ mol L^−1^ s^−1^) was found under oxic conditions, whereas carbamazepine (1.3 × 10^−6^ mol L^−1^ s^−1^) and sulfamethoxazole (1.3 × 10^−6^ mol L^−1^ s^−1^) were only degraded under anoxic aquifer conditions. As Munz et al. (2019) mentioned, there is a possible risk for long-time transport of OMPs like phenazone, diclofenac, and valsartan if the environment is going to be anoxic. This is shown to occur in the second aquifer during the simulation period for phenazone, but in our study, the resulting concentrations were remaining below the health threshold (UBA 2017).

Overall, we recommend a temperature-dependent rate formulation for oxygen and degradation rates with Monod terms to achieve reliable modeling results for OMPs. The use of the temperature constraint for the OMP degradation did not significantly influence our simulation results, except for valsartan. The strongest influence on the general degradation has the maximum rate constant, followed by Monod terms for redox dependencies. The temperature factor acts as a fitting parameter to reduce degradation and thus to adapt the shape of the oxygen breakthrough curve at lower temperatures. Temperature-dependent modeling with a normalized factor could be more praxis-orientated because the temperature is relatively simple to measure and account for but also could be simulated based on measured surface water temperature, as we have outlined. The consideration of the temperature impact could lead to data being better comparable between sites, despite their different boundary conditions and hydrogeological conditions, and help to prevent an underestimation of degradation potential, also for temperatures possibly being somewhat higher in the future.

## Data Availability

Data can be requested from the corresponding author.

## References

[CR1] Arnosti C, Jørgensen BB, Sagemann J, Thamdrup B (1998). Temperature dependence of microbial degradation of organic matter in marine sediments: polysaccharide hydrolysis, oxygen consumption, and sulfate reduction. Mar Ecol Prog Ser.

[CR2] Berk Z (2009) Chapter 19 – Refrigeration, chilling and freezing. In: Berk Z (ed) Food Process Engineering and Technology, 1st edn. Academic Press, pp 391–411. 10.1016/B978-0-12-373660-4.00019-3

[CR3] Burke V, Greskowiak J, Asmuß T, Bremermann R, Taute T, Massmann G (2014). Temperature dependent redox zonation and attenuation of wastewater-derived organic micropollutants in the hyporheic zone. Sci Total Environ.

[CR4] Burke V, Greskowiak J, Grünenbaum N, Massmann G (2017). Redox and temperature dependent attenuation of twenty organic micropollutants – a systematic column study. Water Environ Res.

[CR5] Burke V, Schneider L, Greskowiak J, Zerball-van Baar P, Sperlich A, Dünnbier U, Massmann G (2018). Trace organic removal during river bank filtration for two types of sediment. Water.

[CR6] Canfield DE, Thamdrup B (2009). Towards a consistent classification scheme for geochemical environments, or, why we wish the term ‘suboxic’ would go away. Geobiology.

[CR7] Diem S, Cirpka OA, Schirmer M (2013). Modeling the dynamics of oxygen consumption upon riverbank filtration by a stochastic–convective approach. J Hydrol.

[CR8] Engelhardt I, Prommer H, Moore C, Schulz M, Schüth C, Ternes TA (2013). Suitability of temperature, hydraulic heads, and acesulfame to quantify wastewater-related fluxes in the hyporheic and riparian zone. Water Resour Res.

[CR9] Engelhardt I, Prommer H, Schulz M, Vanderborght J, Schüth C, Ternes TA (2013). Reactive transport of iomeprol during stream-groundwater interactions. Environ Sci Technol.

[CR10] Epting J, Händel F, Huggenberger P (2013). Thermal management of an unconsolidated shallow urban groundwater body. Hydrol Earth Syst Sci.

[CR11] Fenz R, Blaschke AP, Clara M, Kroiss H, Mascher D, Zessner M (2005). Monitoring of carbamazepine concentrations in wastewater and groundwater to quantify sewer leakage. Water Sci Technol.

[CR12] Greskowiak J, Prommer H, Massmann G, Nützmann G (2006). Modeling seasonal redox dynamics and the corresponding fate of the pharmaceutical residue phenazone during artificial recharge of groundwater. Environ Sci Technol.

[CR13] Greskowiak J, Hamann E, Burke V, Massmann G (2017). The uncertainty of biodegradation rate constants of emerging organic compounds in soil and groundwater – a compilation of literature values for 82 substances. Water Res.

[CR14] Grünheid S, Gary A, Jekel M (2005). Removal of bulk dissolved organic carbon (DOC) and trace organic compounds by bank filtration and artificial recharge. Water Res.

[CR15] Hamann E, Stuyfzand PJ, Greskowiak J, Timmer H, Massmann G (2016). The fate of organic micropollutants during long-term/long-distance river bank filtration. Sci Total Environ.

[CR16] Hancock PJ (2002). Human impacts on the stream-groundwater exchange zone. Environ Manag.

[CR17] Harbaugh AW, Banta ER, Hill MC, McDonald MG (2000) MODFLOW-2000, The U.S. Geological Survey Modular Ground-Water Model- user guide to modularization concepts and the groundwater flow processes. Open-File Report 00-92. Reston, Virginia

[CR18] Heberer T, Schmid-Bäumler K, Stan H-J (1998). Occurrence and distribution of organic contaminants in the aquatic system in Berlin. Part I: drug residues and other polar contaminants in Berlin surface and groundwater. Acta Hydrochim Hydrobiol.

[CR19] Heberer T, Massmann G, Fanck B, Taute T, Dünnbier U (2008). Behaviour and redox sensitivity of antimicrobial residues during bank filtration. Chemosphere.

[CR20] Henzler AF, Greskoiwak J, Massmann G (2014). Modeling the fate of organic micropollutants during river bank filtration (Berlin, Germany). J Contam Hydrol.

[CR21] Henzler AF, Greskowiak J, Massmann G (2016). Seasonality of temperatures and redox zonations during bank filtration – a modeling approach. J Hydrol.

[CR22] Huang WE, Oswald SE, Lerner DN, Smith CC, Zheng C (2003). Dissolved oxygen imaging in a porous medium to investigate biodegradation in a plume with limited electron acceptor supply. Environ Sci Technol.

[CR23] Huntscha S, Rodriguez Velosa DM, Schroth M, Hollender J (2013). Degradation of polar organic micropollutants during riverbank filtration: complementary results from spatiotemporal sampling and push−pull tests. Environ Sci Technol.

[CR24] Karam J, Nicell JA (1997). Potential applications of enzymes in waste treatment. J Chem Technol Biotechnol.

[CR25] Kirschbaum MUF (1995). The temperature dependence of soil organic matter decomposition, and the effect of global warming on soil organic storage. Soil Biol Biochem.

[CR26] Lensing HJ, Vogt M, Herrling B (1994). Modeling of biologically mediated redox processes in the subsurface. J Hydrol.

[CR27] Lingens F, Blecher R, Blecher H, Blobel F, Eberspächer J, Fröhner C, Görisch H, Layh G (1985). Phenylobacterium immobile gen. nov., sp. nov., a gram-negative bacterium that degrades the herbicide chloridazon. Int J Syst Bacteriol.

[CR28] Loos R, Locoro G, Comero S, Contini S, Schwesig D, Werres F, Balsaa P, Gans O, Weiss S, Blaha L, Bolchi M, Gawlik BM (2010). Pan-European survey on the occurrence of selected polar organic persistent pollutants in ground water. Water Res.

[CR29] Lu G, Clement TP, Zheng C, Wiedemeier TH (1999). Natural attenuation of BTEX compounds: model development and field-scale application. Ground Water.

[CR30] Margot J, Maillard J, Rossi L, Barry DA, Holliger C (2013). Influence of treatment conditions on the oxidation of micropollutants by *Trametes versicolor* laccase. New Biotechnol.

[CR31] Massmann G, Greskowiak J, Dünnbier U, Zuehlke S, Knappe A, Pekdeger A (2009). The impact of variable temperatures on the redox conditions and the behaviour of pharmaceutical residues during artificial recharge. J Hydrol.

[CR32] McEachran AD, Shea D, Bodnar W, Guthrie Nichols E (2016). Pharmaceutical occurrence in groundwater and surface waters in forests land-applied with municipal wastewater. Environ Toxicol Chem.

[CR33] McMahon PB, Chapelle FH (2008). Redox processes and water quality of selected principal aquifer systems. Ground Water.

[CR34] Moran BN, Hickey WJ (1997). Trichloroethylene biodegradation by mesophilic and psychrophilic ammonia oxidizers and methanotrophs in groundwater microcosms. Appl Environ Microbiol.

[CR35] Munz M, Oswald SE, Schäfferling R, Lensing H-J (2019). Temperature-dependent redox zonation, nitrate removal and attenuation of organic micropollutants during bank filtration. Water Res.

[CR36] O’Connell AM (1990). Microbial decomposition (respiration) of litter in Eucalypt forests of South-Western Australia: an empirical model based on laboratory incubations. Soil Biol Biochem.

[CR37] Park B-H, Bae G-O, Lee K-K (2015). Importance of thermal dispersivity in designing groundwater heat pump (GWHP) system: field and numerical study. Renew Energy.

[CR38] Parkhust DL, Appelo CAJ (1999) User’s guide to PHREEQC (Version 2) – a computer program for speciation, batch-reaction, one-dimensional transport, and inverse geochemical calculations. Water-Resources Investigations Report 99-4259. U.S. Department of the Interior, US Geological Survey

[CR39] Pollock D W (2012) User guide for MODPATH Version 6—a particle-tracking model for MODFLOW: U.S. Geological Survey Techniques and Methods 6–A41, 58 p.

[CR40] Prommer H, Post V (2010) PHT3D. A reactive multicomponent transport model for saturated porous media. User’s Manual v2.10. 10.1111/j.1745-6584.2010.00732.x 20662945

[CR41] Prommer H, Stuyfzand PJ (2005). Identification of temperature-dependent water quality changes during a deep well injection experiment in a pyritic aquifer. Environ Sci Technol.

[CR42] Reddersen K, Heberer T, Dünnbier U (2002). Identification and significance of phenazone drugs and their metabolites in ground- and drinking water. Chemosphere.

[CR43] Rivett MO, Buss SR, Morgan P, Smith JWN, Bemment CD (2008). Nitrate attenuation in groundwater: a review of biogeochemical controlling processes. Water Res.

[CR44] Sadef Y, Poulsen TG, Bester K (2014). Impact of compost process temperature on organic micro-pollutant degradation. Sci Total Environ.

[CR45] Sharma L, Greskowiak J, Ray C, Eckert P, Prommer H (2012). Elucidating temperature effects on seasonal variations of biogeochemical turnover rates during riverbank filtration. J Hydrol.

[CR46] Stonestrom DA. Blasch KW (2003) Determining temperature and thermal properties for heat based studies of surface water ground water interactions: Appendix A of heat as a tool for studying the movement of ground water near streams (Cirl1260), U.S. Geological Survey, 76.

[CR47] Stuyfzand PJ, Segers W, van Rooijen N (2007) Behavior of pharmaceuticals and other emerging pollutants in various artificial recharge systems in the Netherlands. ISMAR6 Proceedings

[CR48] Sui Q, Cao X, Lu S, Zhao W, Qiu Z, Yu G (2015). Occurrence, sources and fate of pharmaceuticals and personal care products in the groundwater: a review. Emerging Contaminants.

[CR49] UBA, Umweltbundesamt (Pub.) (2017) Liste der nach GOW bewerteten Stoffe. https://www.umweltbundesamt.de/sites/default/files/medien/374/dokumente/liste_der_nach_gow_bewerteten_stoffe_201802.pdf. Accessed 6 February 2019

[CR50] Verardo E, Atteia O, Prommer H, Vlassopoulos D (2017) ORTI3D. Open reactive transport interface 3D. User’s manual -1.1.2. For professional application in groundwater flow and reactive transport modelling. By Anchor QEA, G & E and Numineo.

[CR51] Wang W, Oswald SE, Gräff T, Lensing H-J, Strasser D, Munz M (2020). Impact of river reconstruction on groundwater flow during bank filtration assessed by transient 3-D modelling of flow and heat transport. Hydrogeol J.

[CR52] Wiese B, Massmann G, Jekel M, Heberer T, Dünnbier U, Orlikowski D, Grützmacher G (2011). Removal kinetics of organic compounds and sum parameters under field conditions for managed aquifer recharge. Water Res.

[CR53] Zhang W, Li Y, Wang C, Wang P, Hou J, Yu Z, Niu L, Wang L, Wang J (2016). Modeling the biodegradation of bacterial community assembly linked antibiotics in river sediment using a deterministic−stochastic combined model. Environ Sci Technol.

[CR54] Zheng C, Wang P (1999) MT3DMS. A modular three-dimensional multispecies transport model for simulation of advection, dispersion and chemical reactions of contaminants in groundwater systems. Documentation and User’s Guide. Contract Report SERDP-99-. Prepared for U.S. Army Corps of Engineers.

